# Lymph node status in different molecular subtype of breast cancer: triple negative tumours are more likely lymph node negative

**DOI:** 10.18632/oncotarget.15022

**Published:** 2017-02-02

**Authors:** Ning Liu, Zhigang Yang, Xiaozhen Liu, Yun Niu

**Affiliations:** ^1^ Department of Pathology, Bao Di Hospital, Bao Di Clinical College of Tianjin Medical University, Tianjin, China; ^2^ Tianjin Baodi Hospital of Traditional Chinese Medicine, Tianjin, China; ^3^ Tianjin Medical University Cancer Institute and Hospital, National Clinical Research Center for Cancer, Key Laboratory of Cancer Prevention and Therapy of Tianjin, Key Laboratory of Breast Cancer Prevention and Therapy, Tianjin Medical University, Ministry of Education, Tianjin, China

**Keywords:** breast cancer, molecular subtype, triple negative subtype, lymph node status, prognosis

## Abstract

**Background and Objectives:**

To investigate the association between different molecular subtype (MST) and the axillary lymph nodal (ALN) status.

**Materials and Methods:**

A total of 528 female patients with primary breast cancer were collected. Survival estimates were calculated using the Kaplan-Meier method, univariate and multivariate logistic regression models.

**Results:**

Triple negative and Luminal A breast cancers were more frequently node-negative (N0) when compared to Luminal B and Her-2 positive cancers (77.4% and 73.4% vs. 45.3% and 40.0%, respectively; P < 0.0001). We observed a clearly significant difference among ALN status in patients with Her-2 positive (P = 0.001) and Luminal B (P < 0.0001) breast cancer. While no significant prognostic diffreence among different LN status was detected in the Triple negative (P = 0.070) and Luminal A subtype (P = 0.660). On the other hand, we detected no prognostic diffreence among different MST in N1 and N3 subgroups (P = 0.569 and P = 0.484, respectively). Multivariate analysis showed that lymph node status (P < 0.01), molecular subtype (P < 0.01), and tumor size (P < 0.01) were significantly and independently prognostic factors. The c-index of the prognosis nomogram for recurrence prediction was 0.70.

**Conclusion:**

Triple negative breast cancer is not associated more frequently with a higher number of involved nodes. The prognosis nomogram can predict the probability of recurrence patients within 3 or 5 years.

## INTRODUCTION

Axillary lymph nodal (ALN) status is one of the most robust factors correlated to overall survival in breast cancer patients, and as such, it has been an integral component of the staging, prognosis, and treatment of invasive breast cancer [1–3]. Recently, it is well described that breast cancer molecular subtype (MST) are associated with significant differences in prognosis [4–5]. Although several predictors(such as multifocality, higher tumor grade, larger tumor size, and the presence of lymphovascular invasion) of lymph node metastasis have been described [1, 6], the impact of tumor MST on ALN has not been well established [4, 7–8].

Breast cancer composed of at least four major subtypes, namely Luminal A, Luminal B, Her-2 positive and Triple negative breast cancer [4]. Triple negative breast cancer exhibits more aggressive clinical behavior, higher metastatic potential, and poorer prognosis compared to other subtypes, and characterized by an adverse prognosis particularly in case of limited sensitivity against neoadjuvant chemotherapy [9–11]. Despite their aggressive clinical behavior, some studies have shown that lymph node involvement may be less frequent in the Triple negative subtype of breast cancer [12–14].

The aim of this study was to confirm the lower risk of ALN involvement at the time of diagnosis in Triple negative breast cancer patients. In addition, we investigated the association between different molecular subtype and the ALN status in Chinese women diagnosed with primary breast cancer between 2004 and 2009.

## MATERIALS AND METHODS

### Patient selection

We identified 528 cases femal patients with invasive breast cancer diagnosed at two clinical research center (212 cases from Bao Di Clinical College of Tianjin Medical University and 316 cases from Cancer Hospital of Tianjin Medical University, China) from Jan 1, 2004 to Dec 31, 2009. The enrolled patients met the following criterion: (1) breast cancer as the first and only cancer diagnosis, (2) equal to or greater than 10 lymph nodes dissected to ensure adequate nodal clearance, (3) known lymph node size, (4) known ER, PR, Her-2, Ki67, p53 information, (5) no previous neoadjuvant systemic therapy, (6) completed follow-up date during the study period. The exclusion criteria were as following: (1) patients with ductal carcinoma *in situ* (DCIS), (2) patients who received breast conserving surgery, (3) recurrent breast cancer, (4) metastatic breast cancer, (5) patients who underwent neoadjuvant systemic therapy, (6) no sufficient data to allow for the estimation of a hazard ratio (HR) with 95 % confidence intervals (95 % CI).

Lymph node (LN) status was then evaluated based on number of tumor involved axillary lymph node (ALN). A positive node was defined as a lymph node containing any cancer cells by hematoxylin and eosin stain or cytokeratin positivity *via* immunohistochemistry (IHC). Women with micrometastases or macrometastases in the ALNs were considered LN positive. Women with only isolated tumor cells (ITCs) in the ALNs were considered LN negative. In the study period, micrometastases were defined as metastases between 0.2 and 2 mm or a tumor cell count between 10 and 100. In the same period, ALN metastases smaller than 0.2 mm and with a tumor cell count less than 10 were defined as ITCs [15]. According to the above criterion, LN status was divided into four groups: 0 node positive (N0), 1-3 nodes positive (N1), 4-9 nodes positive (N2), more than or equal to 10 nodes positive (N3).

### Immunohistochemical evaluation

The status of estrogen receptor (ER), progesterone receptor (PR), Her-2, Ki67, and p53 was determined by immunohistochemistry (IHC) and collected from pathology reports. IHC was performed using standard procedures. ER and PR were categorized as negative (<1%) and positive (≥1%) of tumor cell nuclear staining, in accordance with recent guidelines [16]. Her-2 was scored for the intensity and the completeness of cell membrane staining based on the 2013 ASCO/CAP guidelines (-, no staining; +, weak partial membranous staining in more than 10 % tumor cells; ++, moderately complete membrane staining in more than or equal to 10 % tumor cells or strong complete membranous staining in less than or equal to 10 % of tumor cells; +++, strong complete membranous staining in more than 10 % of tumor cells). Her-2 (+++) was defined as positive. FISH assay was performed in selected cases (i.e., those with ++ immunoreactivity) [17]. Ki67 status was expressed in terms of percentage of positive cells, with a threshold of 20 % of positive cells [18]. For p53, positive staining of fewer than 10 % of the tumor cells was defined as negative expression and staining of 10 % or more of the tumor cells as positive expression [19]. Because the lymphatic vessels and small veins is difficult to identify, lymphatic vessels and fine veins are collectively tend to referred to as lymphovascular under routine pathological. Lymphatic cancer struck and fine veins around the tumor showed lymphovascular invasion positive.

Based on 2013 St. Gallen Consensus, subtypes of breast cancer (Luminal A, Luminal B, Her-2 positive, and Triple negative) were defined by ER, PR, Ki67, and Her-2 status [18]: Luminal A (ER+ and PR≥20 %, Her-2-, Ki67 < 20 %); Luminal B which include Luminal B-Her-2-negative-like (ER+ and PR-/ < 20 %, Her-2-, Ki67≥20 %), and Luminal B-Her-2-positive-like (ER+ and Her-2+, any PR and Ki67); Her-2 positive (nonluminal: Her-2+, ER- and PR-); Triple negative (basal-like: Her-2-, ER- and PR -).

### Follow-up study and study endpoints

Follow-up data were obtained *via* medical records, making telephone calls and study questionnaire. The primary endpoints were recurrence-free survival (RFS). RFS was calculated as time from surgery to locoregional recurrence (tumor recurrence in the ipsilateral chest wall, axilla, and infraclavicular, supraclavicular, or internal mammary lymph nodes), distant metastasis, or death. The last follow-up date was defined as the last breast cancer evaluation by a physician or a mammogram.

### Statistics

The chi-square test was used to evaluate the relationship between the clinicopathologic variables and the lymph nodes status. The Kaplan-Meier method were used for the RFS analyses. Univariate analyses were performed using Cox proportional hazard models. Multivariate analyses were performed on features including significant clinical and biological features at univariate analysis. Multivariable logistic regression was used to estimate the association of number of positive lymph nodes with a number of variables. Hazard ratio (HR), 95% confidence intervals (CI) and *P*-values were all calculated. All *P*-values were two-sided, and *P*-values < 0.05 were considered to be statistically significant. All analyses were conducted using IBM Statistics SPSS 19.0.

### Nomogram development

The Cox proportional hazards regression model was used to construct the nomogram. The model performance was quantified with respect to discrimination and calibration. Discrimination (i.e., whether the relative ranking of individual predictions is in the correct order) was quantified using the concordance index (c-index). The c-index ranges from 0 to 1, with 1 indicating perfect concordance, 0.5 indicating no better concordance than chance, and 0 indicating perfect discordance [19].

## RESULTS

### Patient characteristics

We included in the analysis 528 women with invasive breast cancer treated between Jan 1, 2004 to Dec 31, 2009. The characteristics of the evaluable patients by lymph node status are given in Table 1. The age of the patients ranged from 18 to 85 years with a mean age and median age of 52.1 years and 51 years, respectively. The N0 consisted of 308 patients (58.3 %), the N1 consisted of 164 patients (31.1 %), the N2 consisted of 35 patients (6.6 %), and the N3 consisted of 21 patients (4.0 %). The breakdown by molecular subtype included 184 (34.8%) patients with Luminal A, 232 (43.9%) with Luminal B, 50 (9.5%) with Her-2 positive, and 62 (11.8%) with Triple negative. Patients with no LN metastasis (N0) had more Triple negative tumors compared to N1, N2, N3 patitnts (15.6% *vs*. 5.5%, 8.6%, and 9.5%; *P* < 0.0001). Patients with N0 had more Luminal A tumors compared to N1, N2, N3 patitnts (43.8% *vs*. 23.8%, 17.1%, and 19.0%). Patients with N3 were more likely to have larger tumor size ( > 5cm; *P* < 0.001), higher tumor grade (G3; *P* = 0.025) when compared to N0, N1, N2 patitnts. Patients with N2 were more likely to have lymphovascular invasion (LVI) (*P* = 0.005). Regarding to treatment, patients with N3 were more likely to received chemotherapy and radiotherapy.

**Table 1:** Clinicopathologic characteristics among different lymph node status

Characteristics		Lymph node status				
	**All**	**N0**	**N1**	**N2**	**N3**	***P*** **value**
	***N***	***N*** **(%)**	***N*** **(%)**	***N*** **(%)**	***N*** **(%)**	
All	528	308 (100.0)	164 (100.0)	35 (100.0)	21 (100.0)	
Molecular subtype						
Luminal A	184	135 (43.8)	39 (23.8)	6 (17.1)	4 (19.0)	
Luminal B	232	105 (34.1)	103 (62.8)	17 (48.6)	7 (33.3)	
Her-2 positive	50	20 (6.5)	13 (7.9)	9 (25.7)	8 (38.1)	
Triple negative	62	48 (15.6)	9 (5.5)	3 (8.6)	2 (9.5)	<0.001*
Tumor size(cm)						
≤2	206	132 (42.9)	60 (36.6)	10 (28.6)	4 (19.0)	
>2; ≤5	252	156 (50.6)	83 (50.6)	10 (28.6)	3 (14.3)	
>5	70	20 (6.5)	21 (12.8)	15 (42.9)	12 (66.7)	<0.001*
Age						
≤50	231	149 (48.4)	60 (36.6)	12 (34.3)	10 (47.6)	
>50	297	159 (51.6)	104 (63.4)	23 (65.7)	11 (52.4)	0.058
Histology						
Ductal	427	259 (84.1)	129 (78.7)	27 (77.1)	12 (57.1)	
Lobular	40	19 (6.2)	14 (8.5)	4 (11.4)	3 (14.3)	
Ductal+Lobular	32	15 (4.9)	11 (6.7)	3 (8.6)	3 (14.3)	
Other	29	15 (4.9)	10 (6.1)	1 (2.9)	3 (14.3)	0.201
Tumor grade						
G1-G2	352	221 (71.8)	100 (61.0)	20 (57.1)	11 (52.4)	
G3	176	87 (28.2)	64 (39.0)	15 (42.9)	10 (47.6)	0.025*
Nodes removed (N)						
10-15	88	51 (16.6)	24 (14.5)	9 (25.7)	6 (21.1)	
>15	440	257 (83.4)	140 (85.5)	26 (74.3)	15 (78.9)	0.405
Lymphovascular invasion						
Yes	226	114 (37.0)	79 (48.2)	22 (62.9)	11 (52.4)	
No	302	194 (63.0)	85 (51.8)	13 (37.1)	10 (47.6)	0.005*
P53						
<10%	340	207 (67.2)	99 (60.4)	22 (62.9)	12 (57.1)	
≥10%	188	101 (32.8)	65 (39.6)	13 (37.1)	9 (42.9)	0.433
Chemotherapy						
Yes	431	239 (77.6)	143 (87.2)	30 (85.7)	19 (90.5)	
No	97	69 (22.4)	21 (12.8)	5 (14.3)	2 (9.5)	0.042*
Radiotherapy						
Yes	145	69 (22.4)	53 (32.3)	13 (37.1)	10 (47.6)	
No	383	239 (77.6)	111 (67.7)	22 (62.9)	11 (52.4)	0.008*
Endocrine therapy						
Yes	286	155 (50.3)	104 (63.4)	19 (54.3)	8 (38.1)	
No	242	153 (49.7)	60 (36.6)	16 (45.7)	13 (61.9)	0.022*
Trastuzumad						
Yes	15	2 (0.6)	2 (1.2)	2 (5.7)	9 (42.9)	
No	513	306 (99.4)	162 (98.8)	33 (94.3)	12 (57.1)	<0.001*

*Difference was statistically significant

### Triple negative tumours are more likely lymph node negative

**Figure 1:**
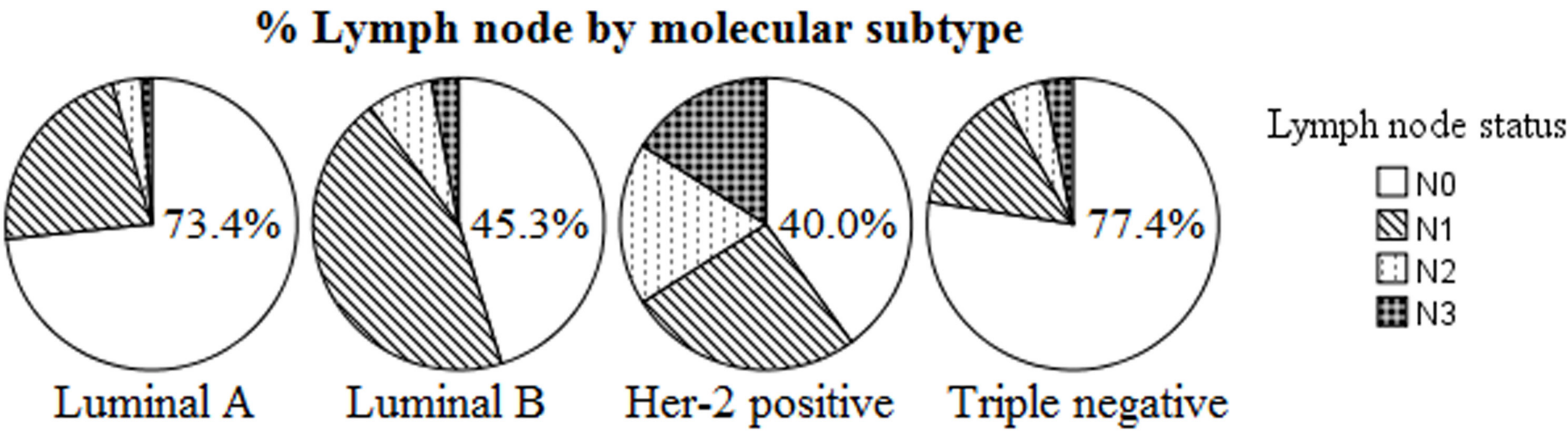
Number of positive lymph nodes by molecular subtype (P < 0.0001) More N0 in Luminal A and Triple negative, more N2 in Her-2 positive and Luminal B.

As shown in Figure 1, Triple negative and Luminal A breast cancers were more frequently node-negative (N0) when compared to Luminal B and Her-2 positive cancers (77.4% and 73.4% *vs*. 45.3% and 40.0%, respectively; *P* < 0.001). On univariate analysis, data suggest that breast cancer subtypes are predictive of lymph node positivity. On multivariable analysis, when adjusted for tumor size, tumor grade, age, and presence of LVI, predictors of LN positivity included Age > 50 (odds ratio [OR] 1.59, 1.03-2.46), presence of LVI (OR 1.47; 1.10-1.95), and tumor size > 5cm (OR 1.62; 1.07-2.44). When compared to the Luminal A subtype, the odds ratio for LN positivity in Triple negative was 0.75, with 95%CI of 0.41-1.40, suggesting that Triple negative breast cancer has nodal involvement less frequently (Table 2).

**Table 2:** Multivariable analysis of predictors of lymph node positivity

Variable	HR	95%CI	*P*-value
Age (>50 vs. ≤50)	1.59	1.03-2.46	0.039*
Tumor grade (G3 vs. G1-G2)	1.14	0.86-1.53	0.362
LVI (Yes vs. No)	1.47	1.10-1.95	0.010*
Tumor size (>2cm;≤ 5cm vs. ≤2cm)	0.92	0.66-1.28	0.604
( >5cm vs. ≤2cm)	1.62	1.07-2.44	0.022*
MST ( Luminal B vs. Luminal A)	1.52	1.04-2.22	0.032*
(Her-2 positive vs. Luminal A)	2.30	1.21-4.38	0.011*
(Triple negative vs. Luminal A)	0.75	0.41-1.40	0.763
(Triple negative vs. Her-2 positive)	0.88	0.59-1.32	0.687

LVI: Lymphovascular invasion; MST: Molecular subtype

*Difference was statistically significant

### The prognostic impact of LN status in different molecular subtypes: Kaplan-Meier plots

**Figure 2:**
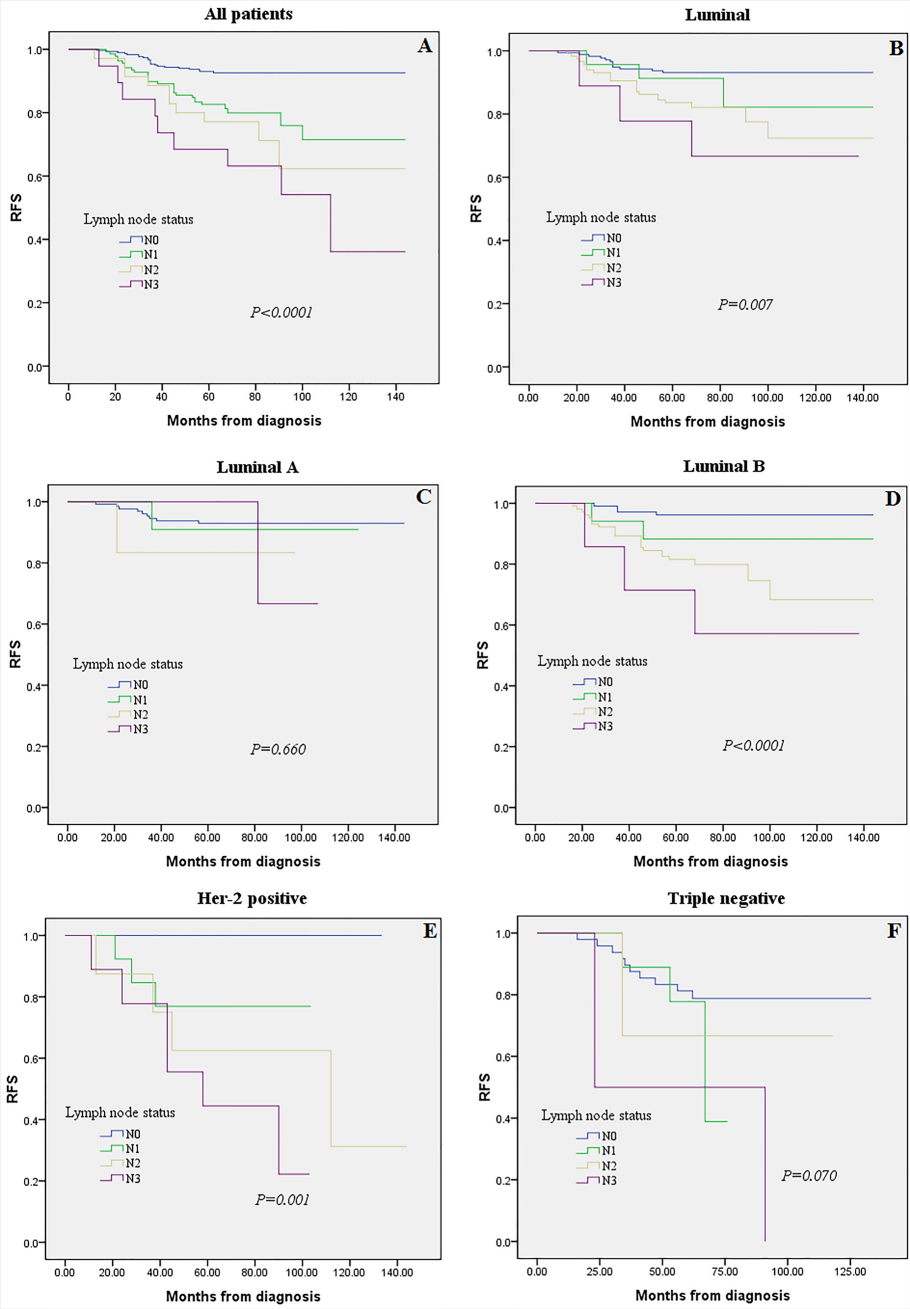
Kaplan-Meier analysis for recurrence-free survival according to lymph node (LN) status were stratified into four groups (N0, N1, N2 and N3) Survival analysis was performed in all 528 patients with follow-up and LNs information **A**., among Luminal tumors **B**., or in the subgroups of Luminal A tumors **C**., Luminal B tumors **D**., Her-2 positive **E**., and Triple negative subtype **F**.

Five hundred and twenty eight patients with complete prognostic information and LN data were subjected to Kaplan-Meier analysis regarding the association between LN status (i.e., N0, N1, N2 and N3) and prognosis (i.e., RFS). Overall, patients showed significant differences in prognosis associated with LN status (Figure 2A, *P* < 0.0001). When restricting the analysis to Luminal group, still a highly significant association of N3 with poor prognosis was retained (Figure 2B, *P =* 0.007). While we detected no significant effect in Luminal A subgroups when analyzing Luminal A and Luminal B separately (Figure 2C, 2D, *P* = 0.660 and *P* < 0.0001, respectively). This effect may be casued by the worse prognosis of Luminal B compared to Luminal A cancers, since the frequency of the Luminal B subtype is nearly doubled in N1 patients (62.8 *vs*. 37.2 %, *P* < 0.001; Table 1). We observed a clearly significant association between LN status among patients with Her-2 positive breast cancer (Figure 2E, *P* = 0.001). In contrast, no significant effect of LN status was detected in the Triple negative subtype (Figure 2F, *P* = 0.070).

### The prognostic impact of molecular subtypes in different LN status: Kaplan-Meier plots

**Figure 3:**
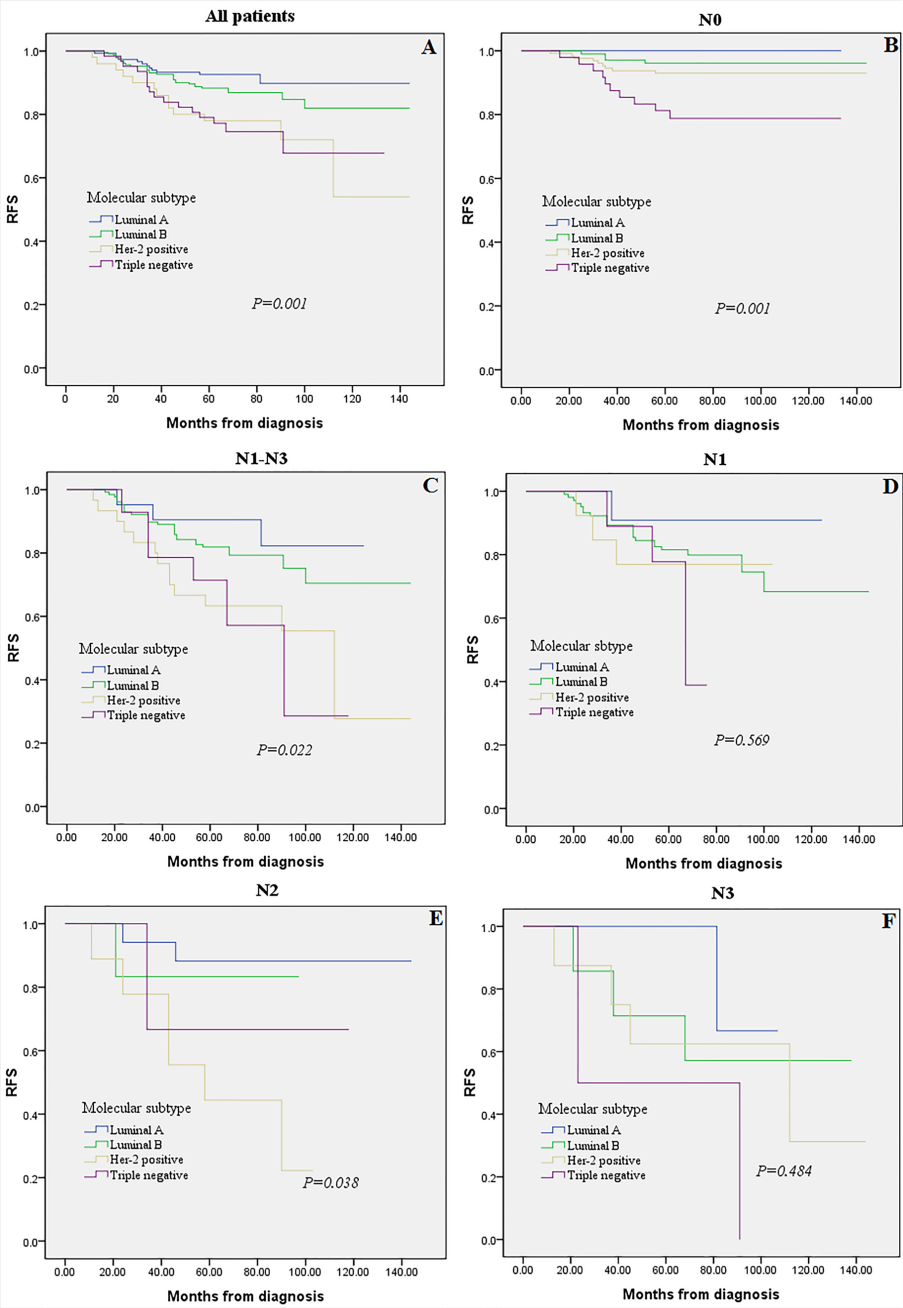
Kaplan-Meier analysis for recurrence-free survival according to molecular subtype Survival analysis was performed in all 528 patients **A**., LN negative(N0) tumors **B**., LN positive(N1-N3) tumors **C**., or in the subgroups of N1 tumors **D**., N2 tumors **E**., and N3 tumors **F**.

As shown in Figure 3, the association between MST (i.e., Luminal A, Luminal B, Her-2 positive, Triple negative) and prognosis (i.e., RFS) was examined. Reduced RFS rates are observed in the Her-2 positive and Triple negative subtypes (Figure 3A, *P* = 0.001). When restricting the analysis to LN negative(N0) and LN positive (N1-N3) group, still a highly significant association of Triple negative breast cancer with poor prognosis was retained (Figure 3B, 3C, *P =* 0.001, *P* = 0.022, respectively). While we detected no significant effect in N1 and N3 subgroups among different MST when analyzing LN positive group separately (Figure 3D, 3F, *P* = 0.569 and *P* = 0.484, respectively).

### The clinicolpathology characteristics and survival: proportional hazards model

We next studied whether the prognostic value that we had observed remains statistically significant in a multivariate analysis in the total cohort. We applied a multivariate Cox regression model which includes lymph node status, molecular subtype of the tumor, tumor size, tumor grade, and lymphovascular invasion. As presented in Table 3, all three, namely, LN status (*P* < 0.01), MST (*P* < 0.01), and tumor size (*P* < 0.01) were significantly and independently prognostic factors in this model.

**Figure 4:**
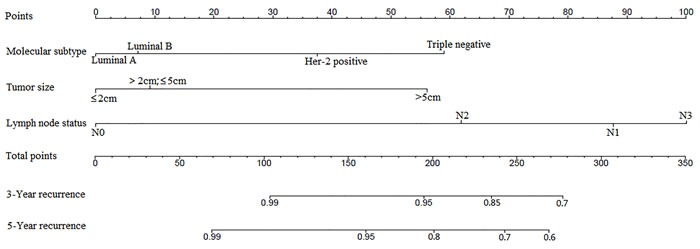
Postoperative nomogram with significant clinicopathologic characteristics predicted the probability of recurrence To use the nomogram, the value attributed to an individual patient is located on each variable axis, and a line is drawn upward to determine the number of points received for each variable value. The sum of these numbers is located on the total points axis, and a line is then drawn downward to the survival axis to determine the 3-year and 5-year LRR likelihood.

To predict the survival of patients, prognostic nomogram was depicted by Cox regression model analysis using all the significant independent indicators for recurrence (Figure 4). The nomogram can predict the probability of recurrence patients within 3 or 5 years. The c-index of the nomogram for recurrence prediction was 0.70.

## DISCUSSION

Breast cancer is a complex, heterogeneous disease at the molecular level. Gene expression studies have identified molecular subtypes (MST) with distinct clinical characteristics in different patients even in different ethnic populations [21]. The immunohistochemistry (IHC) classification of patients has been shown to correlate well with intrinsic classification using gene expression microarrays: ER/PR+, Her-2- with Luminal A; ER/PR+, Her-2+ with Luminal B; ER-, PR-, Her-2+ (Her-2 positive); and ER-/PR-, Her-2- with Triple negative tumors [22, 23]. Triple negative breast cancer has emerged as a group of breast cancer patients with unique therapeutic challenges and worst outcomes, and forms an important area of research interest [24]. Lymph node (LN) status is one of the most robust factors correlated to overall survival in breast cancer patients, and as such, it has been a major determinant in therapeutic decision making [25]. In the present study, we used a cohort of samples with gene expression data obtained from 528 patients with breast cancer to study influence of MST and LN status on prognosis. Our study confirmed the lower risk of axillary lymph nodal (ALN) involvement in Triple negative breast cancer patients. An decreased frequency of LN metastasis were found in Triple negative breast cancer and Luminal A patients (Table 1 and Figure 1). This was not unexpected and in line with several earlier reports [26–28].

Our study demonstrated tumor size as a significant independent predictive factor for positive LN status with an odds ratio of 1.62 for T3 ( > 5cm) *versus* T1 (≤2cm)tumors. The results are in accordance with Viale et al [2] and Silverstein et al [29] analysis, they demonstrated that tumor size to be the most significant predictor of LN metastases. In addition, older patients ( > 50 years old) were 50 % more likely to have positive LNs. With respect to lymphovascular invasion (LVI), a variety of studies have shown strong correlation with ALN involvement. In this study, LVI was a predictor of LN positivity with an odds ratio of 1.47, but the Triple negative phenotype was not associated with LN positivity. However, we found that LN involvement is more frequently observed in Her-2+ tumors (Luminal B and Her-2 positive) as compared to Luminal A and Triple negative subtypes.

Our study has several limitations. Firstly, this study is a retrospective analysis and the number of samples is relatively few. Thus, differences in treatment depending on LN status and molecular subtype at diagnosis cannot be excluded and have the potential to significantly introduce bias into our analyses. A prospective multicentre randomised clinical study should be performed in the future and the results will provide more scientific basis and reliable guidance for judgement of clinical treatment. Second, most patients did not undergo trastuzumab treatment in Her-2 positive patients which has been shown to decrease rates of LRR by 50 % [30]. The reason for lack of adjuvant trastuzumab is that during the period of study enrollment (January 2004 to December 2009), the concept of adjuvant trastuzumab had not been built up completely.

Previously studies suggested that the aggressive nature of Triple negative breast cancer may be due to distant spread in disease early stage [3, 31, 32]. Dent et al [33] showed that patients with Triple negative breast cancer had a greater risk of distant recurrence by means of visceral metastases. Together with the results of our study, it could be hypothesized Triple negative breast cancer has less lymph node metastasis but is more aggressively. It may due to hematogenous spread or lack of targetable treatment. If Triple negative breast cancer is more likely to spread hematogenously, adjuvant systemic treatment might be more beneficial than locoregional radiotherapy and axillary treatment in patients with early TNBC. This requires further investigation to study why triple negative breast cancer have less lymph node metastasis in a serious of clinical and basic research.

In conclusion, we demonstrated that association of LN status with breast cancer MST contributes to its important role as prognostic factor among patients with breast cancer. On the other hand, although Triple negative breast cancer is more aggressive, it does not metastasize more frequently to the axilla and it is not associated more frequently with a higher number of involved nodes. This may indicate that Triple negative breast cancer tends to spread hematogenously rather than lymphogenously, stressing the importance of systemic treatment compared to locoregional treatment in these patients.

### Ethical approval

This study was approved by the Bao Di Clinical College of Tianjin Medical University and Tianjin Medical University Cancer Institute and Hospital, China and has been performed in accordance with the ethical standards laid down in the 1964 Helsinki declaration and its later amendments.

### Synopsis

Our study analyse and compare survival outcomes of breast cancer patients between different molecular subtype and lymph nodes status. Data showed that Triple negative and Luminal A breast cancers were more frequently node-negative. On the other hand, we demonstrated that the nomogram can predict the probability of recurrence patients within 3 or 5 years.
